# Proteomic analysis of differentially expressed proteins in vitamin C-treated AGS cells

**DOI:** 10.1186/1471-2091-14-24

**Published:** 2013-09-26

**Authors:** Arulkumar Nagappan, Hyeon Soo Park, Kwang Il Park, Jin A Kim, Gyeong Eun Hong, Sang Rim Kang, Jue Zhang, Eun Hee Kim, Won Sup Lee, Chung Kil Won, Gon Sup Kim

**Affiliations:** 1 Research Institute of Life Science and College of Veterinary Medicine, Gyeongsang National University, 900 Gajwadong, Jinju, Gyeongnam 660-701, Republic of Korea; 2 Department of Biological Science, Center for Colon Cancer Research, University of South Carolina, Columbia, SC 29208, USA; 3 Korea National Animal Research Resource Center, Korea National Animal Bio-resource Bank, Gyeongsang National University, Gazwa, Jinju 660-701, Republic of Korea; 4 Department of Biological Enginneering, School of Natural Science, Kyonggi University, 14 Yeongtong, Suwon 443-760, Republic of Korea; 5 Key Laboratory of Nuclear Medicine, Ministry of Health, Key Laboratory of Molecular Nuclear Medicine, Jiangsu Institute of Nuclear Medicine, Wuxi, Jiangsu 214063, China; 6 Department of Nursing Science, International University of Korea, Jinju 660-759, Republic of Korea; 7 Department of Internal Medicine, Institute of Health Sciences, Gyeongsang National University School of Medicine, Gyeongnam Regional Cancer Center, Gyeongsang National University Hospital, Jinju 660-702, Republic of Korea

**Keywords:** Vitamin C, Gastric cancer, AGS cells, Proteome analysis, 14-3-3 isoforms

## Abstract

**Background:**

Vitamin C (ascorbic acid) is an essential nutrient of most living tissues that readily acts as a strong reducing agent, which is abundant in fruits and vegetables. Although, it inhibits cell growth in many human cancer cells *in vitro,* treatment in cancer is still controversial. Hence, the purpose of this study was to investigate the molecular mechanism of the inhibitory effect of vitamin C on AGS cell growth, and protein profiles in AGS cells after exposure to vitamin C treatment, by using proteomic tools.

**Results:**

Vitamin C showed a cytotoxic effect on AGS cells (IC50 300 μg/mL) and, 20 differentially expressed proteins (spot intensities which show ≥2 fold change and statistically significant, p<0.05 between the control and vitamin-C treated group) were successfully identified by assisted laser desorption/ ionization-time of flight/mass spectrometry (MALDI-TOF/MS). Of the 20 proteins, six were up-regulated and fourteen were down-regulated. Specifically, 14-3-3σ, 14-3-3ϵ, 14-3-3δ, tropomyosin alpha-3 chain and tropomyosin alpha-4 chain were down-regulated and peroxiredoxin-4 and thioredoxin domain-containing proteins 5 were up-regulated. The identified proteins are mainly involved in cell mobility, antioxidant and detoxification, signal transduction and protein metabolism. Further, the expressions of 14-3-3 isoforms were verified with immuno-blotting analysis.

**Conclusions:**

Our proteome results suggest that the apoptosis related proteins were involved in promoting and regulating cell death of AGS cells, and might be helpful to understand the molecular mechanism of vitamin C on AGS cell growth inhibition.

## Background

Globally, gastric cancer is the second leading cause of cancer-related death, and is the most prevalent cancer in South Korea. In the past two decades, the mortality and incidence of gastric cancer has decreased gradually but it is still the second most common cancer in Asia [1]. Most stomach cancers are an adenocarcinoma type, which accounts approximately 90% [2]. The well-established risk factors are Helicobacter pylori-infection and cigarette smoking, and the role of dietary factors has also been suggested [3]. The most widely used treatments for stomach cancer are surgery, chemotherapy, and/or radiation therapy. The available treatments are not effective and recuperation is also still problematic. Hence, there is an urgency to apply new therapeutic agents to increase survival rates of gastric cancer patients.

Vitamin C (ascorbic acid) is an essential nutrient of most living tissues, and readily acts as a strong reducing agent [4]. Epidemiological studies have reported that vitamin C deficiency in humans are linked to more severe *H. pylori*-associated gastritis and a gastric cancer risk is also higher [5]. The study reported that supplementation of vitamin C has been associated with reduced gastric cancer risk in humans. In addition, a reduced risk for most types of cancer is associated with a high intake of fresh fruits and vegetables, which contain vitamin C [6]. Despite, the controversial cancer treatment history, the *in-vitro* studies reported that ascorbate induces cell cycle arrest and apoptosis in various tumor cells [7,8]. However, the exact mechanism of vitamin C involved in cancer treatment is not fully elucidated.

A global proteomic approach is being extensively applied in cancer research [9]. This approach uses a combination of two-dimensional gel electrophoresis (2-DE), image analysis, matrix-assisted laser desorption/ ionization-time of flight (MALDI-TOF) mass spectrometry (MS), and bioinformatics analyses to comprehensively resolve, identify, and characterize proteins in the cells, tissues and animal models. These high-throughput proteome techniques allow us to examine the changes in protein expression of AGS cells in response to vitamin C. Identification of differentially expressed proteins is important to understand the molecular events involved in vitamin C anti-cancer mechanism and protective effects, as well as brings new insights into AGS carcinogenesis. Regarding gastric cancer, proteome analysis has been reported mainly in KATO-III and EPG 85–257 human gastric cancer cell lines [10,11]. 2-DE maps have also permitted to obtain an overview of the expressed proteins in the human stomach [12]. Also, proteome analysis has been carried out in 11 human gastric cancer samples to find the biomarkers of gastric cancer [13]. However, no 2-DE proteome of vitamin C-treated AGS cells have hitherto been reported.

Our previous study demonstrated that vitamin C induced apoptosis in human adenocarcinoma AGS cells at pharmacological concentrations, and inhibited AGS cells proliferation [14]. In the present study, we perform a proteome analysis of AGS cells treated with vitamin C at pharmacological concentrations (300μg) and the control (only vehicle), and 20 different expressed proteins were identified by MALDI-TOF/MS. Also, the expression of isoforms of 14-3-3 proteins was confirmed by immuno-blotting. The cytotoxicity assay suggests that vitamin C inhibited AGS cells growth and proteome results revealed that apoptosis related proteins were involved in promoting and regulating cell death of AGS cells.

## Methods

### Chemical and reagents

RPMI 1640 medium was purchased from Hyclone (Logan, UT, USA). Fetal bovine serum (FBS) and antibiotics (streptomycin/penicillin) were purchased from Gibco (BRL Life Technologies, Grand Island, NY, USA). Materials and chemicals used for electrophoresis were obtained from BioRad (Hercules, CA, USA). Antibody to 14-3-3σ and β-actin were purchased from Millipore (Billerica, MA, USA). 14-3-3ϵ and 14-3-3δ were obtained from Bioworld Technology Inc. (St. Louis Park, MN, USA)). Vitamin C was provided by Animal Resources Research Bank (ABRB). All other chemicals used in this study were purchased from AMRESCO (Solon, OH, USA) and Sigma-Aldrich (St. Louis, MO, USA). All the chemicals used were of the highest grade commercially available.

### Cell culture and treatments

AGS human gastric cancer cell line was purchased from ATCC (Manassas, VA, USA). Cells were grown in RPMI 1640 medium supplemented with 10% FBS and 1% penicillin/streptomycin (P-S), and grown in a humidified incubator with 5% CO_2_ in air at 37°C. Experiments were performed when cell growth was approximately 80% confluent.

### Cytotoxicity assay

The 3-(4, 5-dimethythiazol-2-yl)-2, 5-diphenyltetrazolium bromide (MTT)-based assay was performed to determine the cytotoxicity of vitamin C on AGS cells. Cells were seeded at 10 × 10^4^ cells/mL in a 12-well plate and incubated for 24 h. Cells were treated with various concentrations of vitamin C (100, 200, 300, 400, and 500 μg/mL) or only vehicle (1X PBS used as the control) and incubated for 24 h. After incubation, 100 μl of a MTT solution (5 mg/mL in 1X phosphate buffered saline, PBS) was added to the wells and incubated for 3 h. Then, 500 μl of dimethyl sulfoxide (DMSO) was added to each well after the medium was removed completely to dissolve the cellular crystalline deposits. The optical density was measured at 540 nm using an ELISA plate reader.

### Protein extraction and two-dimensional gel electrophoresis

A total of 1×10^7^ cells was plated onto 100mL plates and incubated overnight at 37°C in an atmosphere of 5% CO2. Cells were treated with 300 μg/mL of vitamin C and 1X PBS used as the control. After 24 h incubation, cells were trypsinized and washed twice with cold 1X PBS. Then, cells were lysed in a lysis buffer (7 M urea, 2 M thiourea, and 4% (w/v) CHAPS) on ice for 1 h. The lysates were centrifuged at 14000 rpm for 15 min at 4°C, and the collected supernatant was stored at −80°C until analysis. Proteins in lysates were precipitated with equal volume (1:1) of 20% v/v trichloroacetic acid and dissolved in 7 M urea, 2 M thiourea, and 4% (w/v) CHAPS, 0.5% (v/v) IPG buffer, and 1% dithiothreitol (DTT). Protein concentration was determined by the Non-Interfering™ protein assay kit (G-Biosciences, St. Louis, MO, USA), in accordance to the manufacturer’s protocol. Immobilized 18 cm linear pH gradient (IPG) strips, pH 4–7, were rehydrated in a rehydration buffer (7 M urea, 2 M thiourea, 4% (w/v) CHAPS, 0.002% Bromophenol blue). For the first dimension, 100 μg protein was focused using the Ettan IPG Phor II (GE Healthcare) at 50 V for 1 h, followed by 200 V for 1 h, 500 V for 30 min, 4000 V for 30 min, 4000 V for 1 h, 10000 V for 1 h, 10000 V for 13 h, and 50 V for 3 h. The focused strips were equilibrated twice, 15 min each time, first with 10 mg/mL DTT and then with 40 mg/mL iodoacetamide (IAA) prepared in equilibration buffer containing 50 mM Tris–HCl (pH 8.8), 6 M urea, 30% (v/v) glycerol, 2% (w/v) SDS, and 0.002% (w/v) Bromophenol blue. The focused proteins were then separated in the second dimension by 12% linear gradient SDS-PAGE with a constant current of 20 mA/gel at 20°C. Gels were run until the Bromophenol dye front reached the end of the gel.

### Protein detection, analysis, and in-gel digestion

The gels were stained with silver nitrate, similar to the method described by Swain and Ross [15] with slight modifications. Three independent gels were performed in triplicate. Gels were scanned and image analysis was performed, using Progenesis Samespots software (Nonlinear Dynamics, Newcastle, UK). Using this software, the differentially expressed spots were identified by automatic matching of the detected protein spots. Those spots differing significantly (p<0.05) in their intensities with a fold-change ≥2 were used for further analysis. Selected protein spots were excised manually from the two-dimensional electrophoresis (2-DE) gel and protein digestion was performed [16] with slight modifications. Briefly, the excised gel pieces were washed with 100 μl of 100 mM NH_4_HCO_3_ for 5 min, and then dehydrated in 100 μl of acetonitrile for 10 min. After being dried in a lyophilizer (SFDSM06, Samwon Freezing Engineering Co., Busan), the gel pieces were rehydrated in 5–10 μl of 50 mM NH_4_HCO_3_ containing 20 ng/μl trypsin (Promega, Madison, WI, USA) on ice. After 45 min, the trypsin solution was removed and replaced with 10–20 μl of 50 mM NH_4_HCO_3_ without trypsin, and digestion was carried out for a minimum of 16 h at 37°C. These peptide mixtures were collected and analyzed by a mass spectrometry.

### Matrix-assisted laser desorption/ionization-time of flight mass spectrometry (MALDI-TOF MS) mass spectrometry and database searching

Tryptic peptides obtained as described above were subsequently extracted by an addition of 10 μl of the extraction buffer, followed by an addition of 10–15 μl of acetonitrile. Pooled extracts were dried in a lyophilizer (SFDSM06, Samwon Freezing Engineering Co., Busan) and the extracts were re-dissolved in 1 μl of extraction buffer and 1 μl of matrix solution (α-acyano- 4-hydroxycinnamic acid, HCCA) and targeted onto a MALDI-TOF plate. After drying the samples completely onto the targeting plate, MALDI-TOF/MS was conducted using a Voyager- DE STR mass spectrometer (Applied Biosystems, Franklin Lakes, NJ, USA) equipped with delay ion extraction. Mass spectra were obtained over a mass range of 800–3,000 Da. For identification of proteins, the peptide mass fingerprinting data were used to search against the Swissprot database using the Mascot program (http://www.matrixscience.com). The following parameters were used for database searches: taxonomy, *Homo sapiens* (human); cleavage specificity, trypsin with one missed cleavage allowed; peptide tolerance of 100 ppm for the fragment ions; and allowed modifications, Cys Carbamidomethyl (fixed), and oxidation of Met (variable). Protein scores >56 were considered statistically significant (*p*<0.05).

### Western blot analysis

AGS cells were cultured in 6-well plates and incubated with vitamin C at 300 μg/mL or PBS as the solvent control for 24 h. After incubation, cells were washed with ice-cold PBS and lysed with a lysis buffer [50 mM Tris–HCl (pH 8.0), 150 mM NaCl, 0.5% sodium deoxycholate, 0.1% sodium dodecyl sulfate (SDS) and 1% NP-40], containing the protease inhibitor cocktail. The cell debris was removed by centrifugation at 13,000 rpm for 30 min and protein concentration was determined using a Bradford assay (Bio-Rad). Proteins were separated by 10% SDS-polyacrylamide gel electrophoresis (SDS-PAGE) and transferred to a polyvinyldene fluoride (PVDF) membrane (Immunobilon-P, 0.45 mm; Millipore) using the TE 77 Semi-Dry Transfer Unit (CE Healthcare Life Sciences, Buckinghamshire, UK). The membrane was blocked with 5% non-fat skim milk in Tris-buffered saline containing 1% Tween-20 (TBS, pH 7.4) at room temperature for 1 h, and the blots were probed with rabbit monoclonal antibody to 14-3-3σ, 14-3-3ϵ and 14-3-3δ, and mouse monoclonal antibody for β-actin. The proteins were visualized using an enhanced chemiluminescence kit (ECL) and Western blotting detection reagents (GE Healthcare Life Sciences), and exposed to X-ray film (Fuji, Tokyo, Japan). Each band was quantitatively determined using Image J (http://rsb.info.nih.gov) software. The densitometry readings of the bands were normalized to β-actin expression.

### Statistical analysis

The data represents the mean±standard deviation (SD) of three independent experiments. The statistical significance between the control and sample groups was calculated by the Student’s t-test. A *p* value <0.05 was considered as significant.

## Results

### Growth inhibition of AGS cells by vitamin C

**Figure 1:**
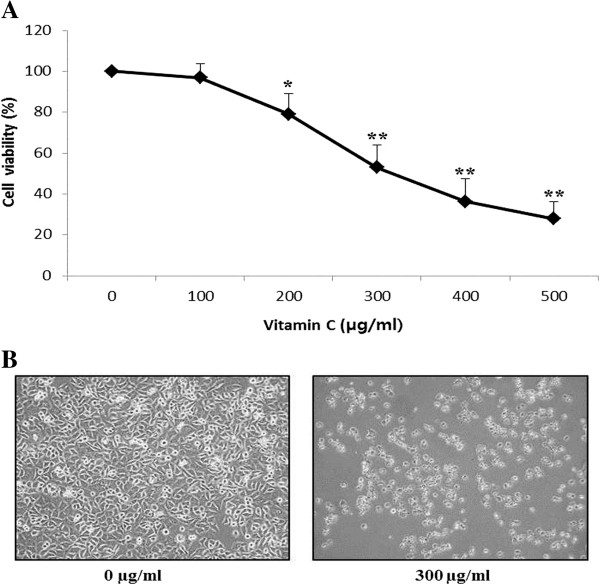
**Effects of vitamin C on cell viability on AGS adenocarcinoma gastric cancer cell line. (A)** The cells were exposed to vitamin C at the indicated dosages for 24 h and cell viability was determined. Vehicle-treated cells were arbitrarily set as 100% control viability. **(B)** Morphology of AGS cells treated with or without vitamin C for 24 h and examined by light microscopy (400). Results are expressed as the mean ± SD of three independent experiments (*P < 0.05; **P < 0.01, compared to control).

To evaluate the effects of growth inhibition and survival of AGS cells, the AGS cells were cultured in the presence of various concentrations (0-500 μg/mL) of vitamin C for 24 h. Vitamin C had a strong inhibitory effect on cell proliferation of AGS cells in a dose-dependent manner when compared to the control, after 24 h treatment with vitamin C (Figure 1A). Especially, vitamin C at 300, 400 and 500 μg/mL decreased the cell growth by approximately 50%, 36% and 27%, respectively. Therefore, the IC50 (50% inhibitory concentration) of vitamin C was found to be approximately 300 μg/mL. Moreover, microscopic observations revealed morphological changes in AGS cells, such as cell shrinkage and density compared with the control cells (Figure 1B). Further, 2-DE gel analysis was performed to study the protein expressions in AGS cells due to inhibitory effects of vitamin C.

### Proteomic analysis to identify differentially expressed proteins in vitamin C-treated AGS cells

**Figure 2:**
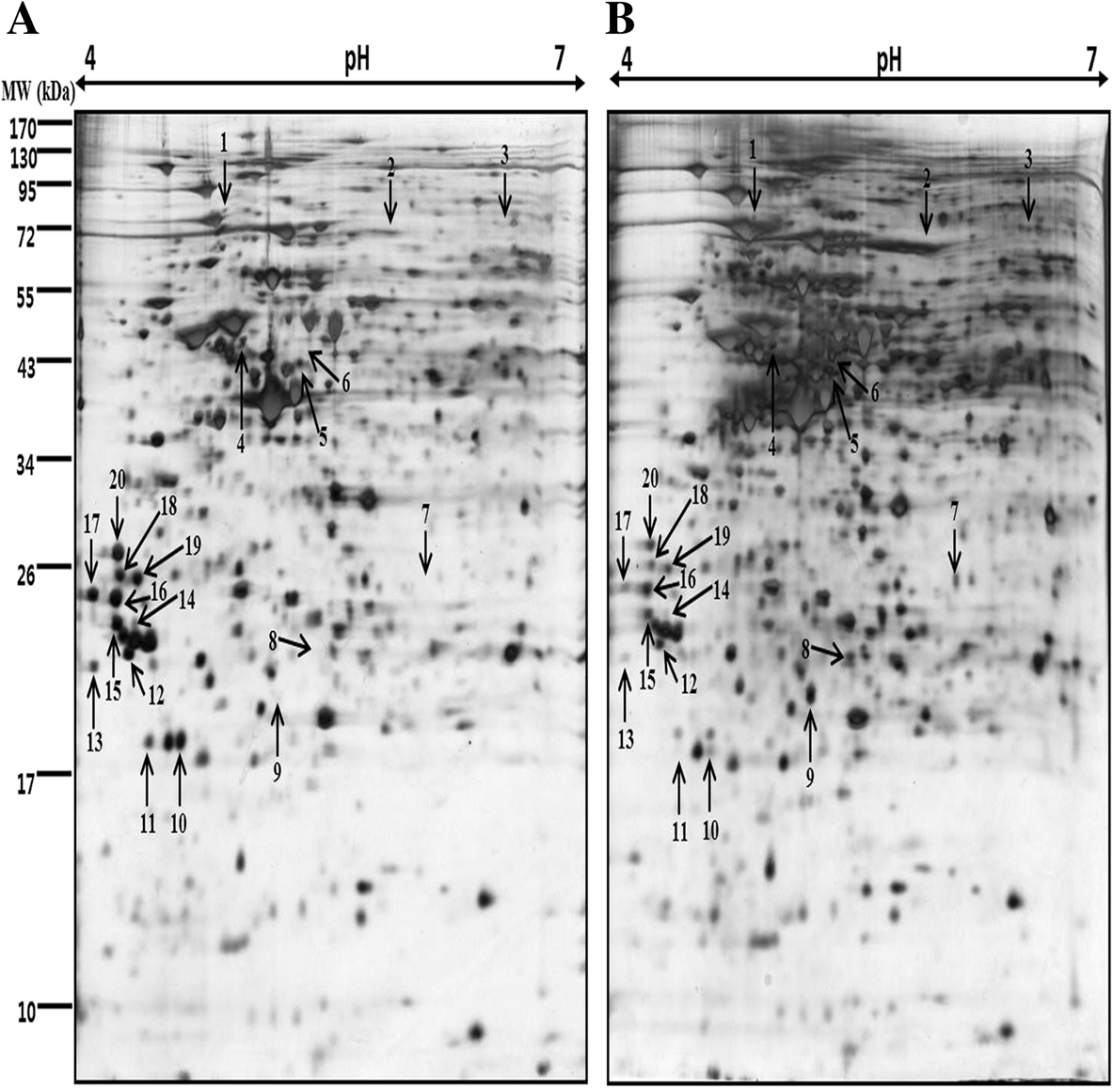
**Two-dimensional map of proteins from AGS gastric cancer cells treated with vitamin C.** Proteins were isolated after exposure of the cells to **A)** control (only vehicle) and **B)** 300 μg/ml of vitamin C for 24 h and separated on IPG-strips with pH 4–7 in the first dimension, and then on 12% polyacrylamide gel on second dimension. The gels were silver stained.

We performed a proteomic approach to identify proteins that were differentially expressed in vitamin C treated (300 μg/mL) AGS cells, 100 μg of total proteins were separated by IEF on 18 cm IPG strips in the first dimension and 12% SDS-PAGE in the second dimension. We observed a total of approximately 500 protein spots in silver stained gels. Control (only vehicle) and vitamin C treated gels were analyzed by using Progenesis Samespots software (Nonlinear Dynamics, Newcastle, UK), and we found 32 statistically significant differentially expressed protein spots (≥2-fold and p<0.05). These 32 differentially expressed proteins spots were chosen for further analysis by MALDI-TOF/MS. Finally, 20 differentially expressed proteins were successfully identified by using the MASCOT search engine and the SwissProt database (Figure 2 and Table 1).

**Table 1:** Differentially expressed proteins in Vitamin C treated AGS cells identified by MALDI-TOF/MS

**Spot no.**	**Swissprot entry name**^**1**^	**Protein name**^**1**^	**Accession number**^**1**^	**Theoretical/ experimental Mr (kDa)**^**2**^	**Theoretical/ experimental pI value**^**2**^	**Protein score**^**3**^	**Sequence coverage (%)/peptides matched**	**Fold change**
**Up - regulated proteins**								
2	TNIP2_HUMAN	TNFAIP3-interacting protein 2	Q8NFZ5	49.24/91	6.03/5.83	56	15/7	2.3
3	XIRP1_HUMAN	Xin actin-binding repeat-containing protein 1	Q702N8	199.64/96	5.78/6.48	59	11/13	2.2
6	TXND5_HUMAN	Thioredoxin domain-containing protein 5	Q8NBS9	48.28/49	5.63/5.38	81	22/10	2.9
7	CE290_HUMAN	Centrosomal protein of 290 kDa	O15078	290.89/28	5.75/6.04	69	9/16	8.5
8	PRDX4_HUMAN	Peroxiredoxin-4	Q13162	30.75/24	5.86/5.43	68	38/6	3.0
9	CE290_HUMAN	Centrosomal protein of 290 kDa	O15078	290.89/23	5.75/5.21	60	8/15	6.4
**Down - regulated proteins**								
1	DPP3_HUMAN	Dipeptidyl peptidase 3	Q9NY33	82.88/100	5.02/4.86	110	26/12	4.0
4	DCTN2_HUMAN	Dynactin subunit 2	Q13561	44.32/51	5.10/4.94	122	39/13	2.3
5	IF4A1_HUMAN	Eukaryotic initiation factor 4A-I	P60842	46.35/48	5.32/5.31	175	47/17	2.1
10	PSB6_HUMAN	Proteasome subunit beta type-6	PSMB6	25.57/21	4.80/4.64	104	39/10	4.4
11	EIF3K_HUMAN	Eukaryotic translation initiation factor 3 subunit K	Q9UBQ5	25.33/21	4.81/4.46	101	37/8	3.6
12	PSA5_HUMAN	Proteasome subunit alpha type-5	P28066	26.57/24	4.74/4.31	91	41/8	2.0
13	IF6_HUMAN	Eukaryotic translation initiation factor 6	P56537	27.10/23	4.56/4.13	86	50/7	3.8
14	1433Z_HUMAN	14-3-3 protein zeta/delta	P63104	27.9/25	4.73/4.36	93	40/10	2.6
15	1433S_HUMAN	14-3-3 protein sigma	P31947	27.87/25	4.68/4.26	150	58/12	2.2
16	1433E_HUMAN	14-3-3 protein epsilon	P62258	29.33/26	4.63/4.27	131	93/12	2.3
17	EF1B_HUMAN	Elongation factor 1-beta	P24534	24.92/26	4.50/4.12	130	50/11	3.3
18	TPM4_HUMAN	Tropomyosin alpha-4 chain	P67936	28.62/27	4.67/4.28	94	25/10	3.1
19	TPM3_HUMAN	Tropomyosin alpha-3 chain	P06753	32.86/27	4.68/4.37	95	20/11	2.7
20	PCNA_HUMAN	Proliferating cell nuclear antigen	P12004	29.09/29	4.57/4.28	127	40/10	2.5

^1^Entry name, protein name and accession number from SWISS-PROT database identified by MALDI-TOF/MS.

^2^Theoretical molecular weight (kDa) and pI from SWISS-PROT database; the experimental values of pI and MW for every isoelectric spot were calculated using Progenesis samespots software (Nonlinear Dynamics Ltd., Newcastle, UK).

^3^Score is −10*log (p), where p is the probability that the observed match is a random event, Protein scores greater than 56 are significant (p<0.05).

**Figure 3:**
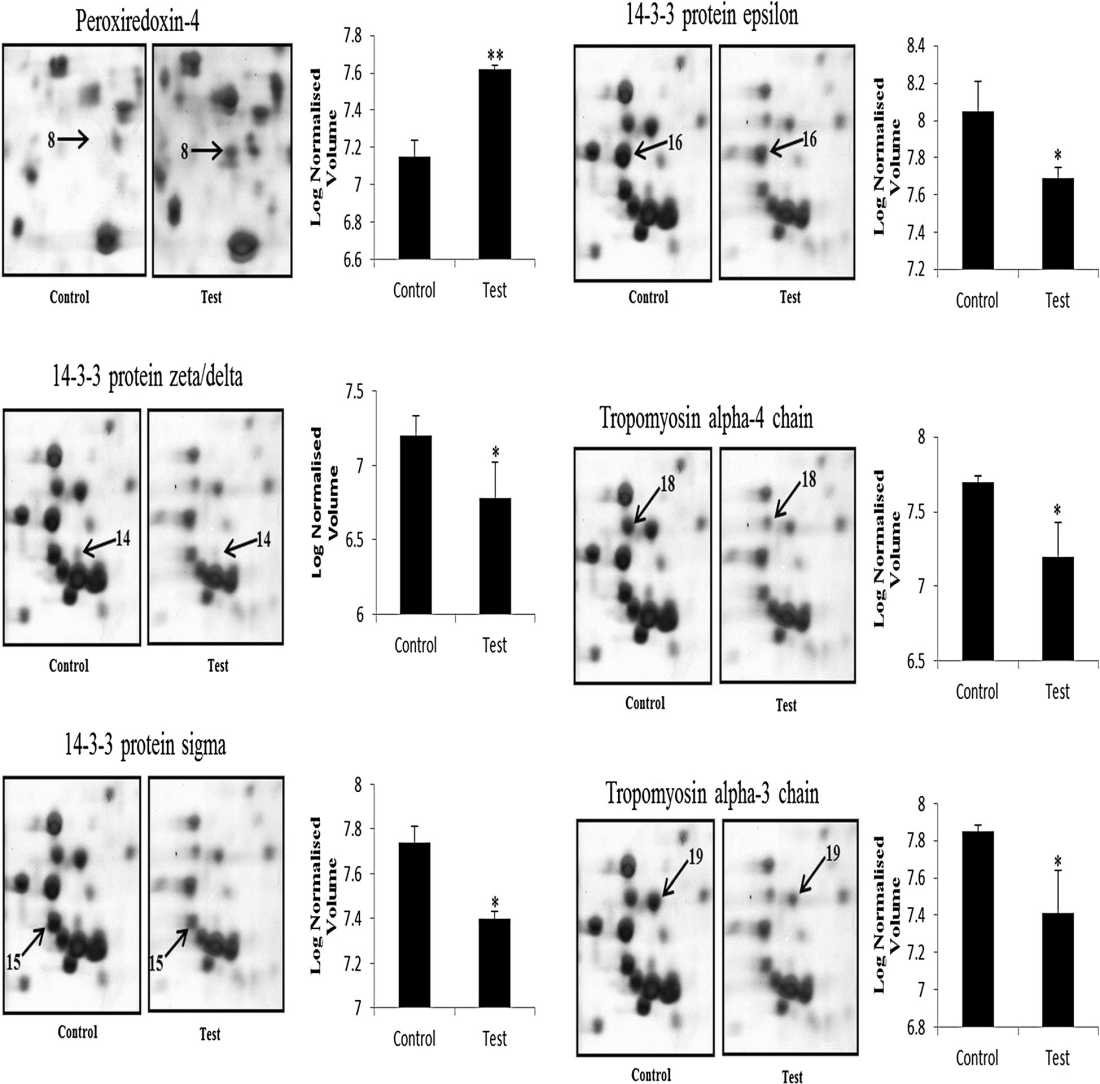
**Enlargements of the 2-DE map of proteins from AGS gastric cancer cells treated with vitamin C, which displays corresponding sections of gels with protein spots derived from control and vitamin C treated cells.** Three independent experiments were performed and the mean ± SD was plotted (*P < 0.05, **P < 0.01 compared with control).

Of these 20 proteins, six were up-regulated and fourteen were down-regulated in vitamin C-treated AGS cells compared with the control. Down-regulated proteins involved in cell motility included tropomyosin alpha-3 chain (TPM3) and tropomyosin alpha-4 chain (TPM4), whereas Xin actin-binding repeat-containing protein 1 (XIRP1) was up-regulated. In addition, the peroxiredoxin-4 (PRDX4) and thioredoxin domain-containing protein 5 (TXND5) were involved in antioxidant and detoxification, which was up-regulated. While proteins participating in signal transduction were significantly down-regulated, including 14-3-3 protein sigma (SFN), 14-3-3 protein epsilon (YWHAE) and 14-3-3 protein zeta/delta (YWHAZ), whereas TNFAIP3-interacting protein 2 (TNIP2) was up-regulated. Proteins involved in protein metabolism Eukaryotic translation initiation factor 3 subunit K (EIF3K), Proteasome subunit alpha type-5 (PSMA5) and Proteasome subunit beta type-6 (PSMB6) were down-regulated. Further, we showed the enlarged 2-DE images of 6 important protein spots, one spot (Peroxiredoxin-4) was up-regulated and the other five (14-3-3σ, 14-3-3ϵ, 14-3-3δ, tropomyosin alpha-3 chain and tropomyosin alpha-4 chain) were down-regulated in the vitamin C-treated AGS cells compared with the control (Figure 3).

### Validation of expression of 14-3-3 isoforms by immunoblotting

**Figure 4:**
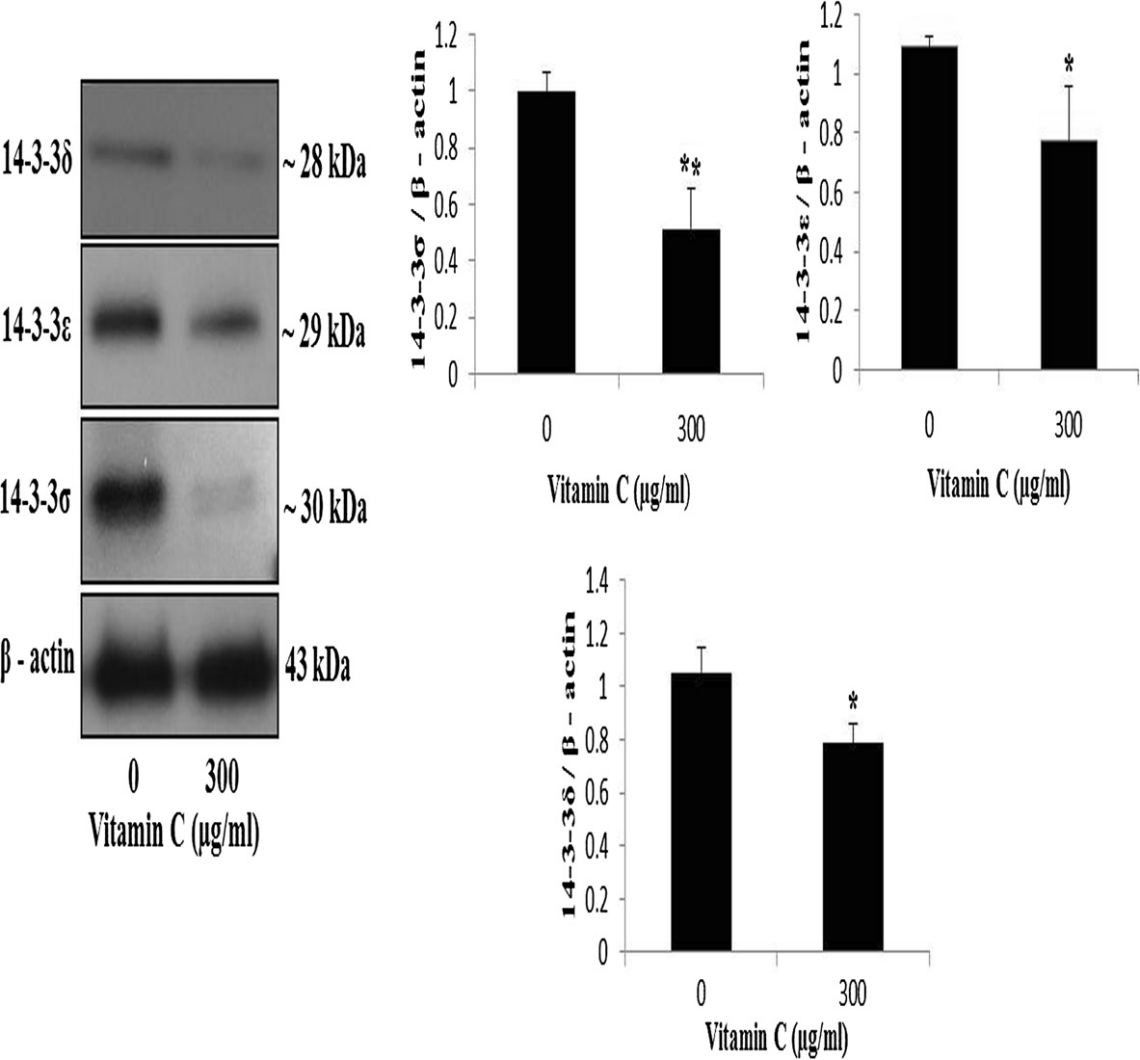
**Effects of vitamin C on expression of 14-3-3 isoforms in AGS cells.** Cells were treated with vitamin C (0 and 300 lg/ml) for 24 h. The cell lysates were subjected to SDS–PAGE and analyzed by Western blotting. Densitometric analyses of data were expressed as the mean ± SD of three independent experiments (*P < 0.05, **P < 0.01 compared with control).

Recent research on cancer targets have focused 14-3-3 proteins that are known to be involved in various biological processes like signal transduction, cell cycle control, apoptosis, cellular metabolism, proliferation, cytoskeletal regulation, transcription, and redox-regulation or stress response. The AGS cells were treated with vitamin C (0, and 300 μg/mL) and the expression of 14-3-3σ, 14-3-3ϵ and 14-3-3δ were examined by immuno-blotting. Quantification of the protein bands revealed that the expression of 14-3-3σ, 14-3-3ϵ and 14-3-3δ were decreased in the vitamin C-treated group compared to the vehicle-treated control group (Figure 4). These data indicated that vitamin C decreased the expression of 14-3-3 isoforms (14-3-3σ, 14-3-3ϵ and 14-3-3δ) in AGS cells.

## Discussion

Apart from antioxidant activity, vitamin C plays an effective role of cancer prevention and treatment. The numerous studies have reported that vitamin C prevents cell proliferation and metastasis of many human cancer cells [17-19]. But, it’s exact molecular mechanisms still has not been fully elucidated. In the previous study, we demonstrated that vitamin C at pharmacological concentration induced apoptosis in AGS cells, mainly through the down-regulation of 14–3-3σ protein and dephosphorylation Bad proteins via a mitochondrial dependent pathway [14]. In the present study, we performed 2-DE analysis coupled with MALDI-TOF/MS of AGS cells treated with vitamin C at a pharmacological concentration (300 μg/mL) and the control (only vehicle), and 20 differentially expressed proteins were identified. Proteomic analysis revealed that the apoptosis related proteins were involved in promoting and regulating cell death of AGS cells.

Ascorbic acid is an excellent antioxidant and ascorbate caused toxicity to cancer cells, but had no effect on normal cells at the same concentration [20]. In the present study, vitamin C had a strong inhibitory effect on cell proliferation of AGS cells in a dose-dependent manner after 24 h treatment with vitamin C (Figure 1A), and the IC50 of vitamin C was found approximately 300 μg/mL or 1.7 mM/mL. And also, morphological changes were observed in AGS cells, such as cell shrinkage and density in vitamin C treated cells compared with the control cells (Figure 1B). This result revealed that vitamin C inhibited AGS cell growth at pharmacological concentrations. Further, 2-DE gel analysis was performed to study the protein expressions in AGS cells due to inhibitory effects of vitamin C. The silver stained gels of control (only vehicle) and vitamin C treated gels were analyzed by using Progenesis Samespots software (Nonlinear Dynamics, Newcastle, UK), and we found 32 statistically significant differentially expressed protein spots (≥2-fold and p<0.05). Finally, 20 differentially expressed proteins were successfully identified by MALDI-TOF/MS analysis using the MASCOT search engine and the SwissProt database (Figure 2 and Table 1). Among 20 proteins, six were up-regulated and fourteen were down-regulated in vitamin C-treated AGS cells compared with the control. These proteins are mainly involved in cell mobility, antioxidant and detoxification, signal transduction and protein metabolism.

### Vitamin C down-regulated proteins involved in the signal transduction, 14-3-3 isoforms

Research on cancer targets have determined that 14-3-3 proteins are known to be involved in various biological processes like signal transduction, cell cycle control, apoptosis, cellular metabolism, proliferation, cytoskeletal regulation, transcription, and redox-regulation or stress response [21]. Among these differentially expressed proteins, three isoforms of 14-3-3 proteins, 14-3-3σ and 14-3-3ϵ and 14-3-3δ were down-regulated (Figure 2). The Bad protein, a proapoptotic family member, is one of the targets of 14-3-3 proteins [22]. When Bad disassociated from 14-3-3, the Bad is found localized to the mitochondria bound to Bcl-2 and Bcl-xL, and induced cell death [23,24]. In addition, vitamin C induced apoptosis by down-regulation of 14–3-3σ and dephosphorylation of Bad via a mitochondrial dependent pathway in AGS cells [14]. Moreover, the remarkable dissociation of Bad from 14-3-3β is the apoptosis mechanism of vitamin C through the increasing of ER stress and the translocation of Bad to mitochondria after dissociation from 14-3-3β in human colon cancer cell line, HCT-8 [25]. These findings suggest that Bad dissociated from 14-3-3 is a key mediator in vitamin C-induced apoptosis through the disruption of mitochondrial membrane potential.

The down-regulation of 14-3-3σ protein has been reported in many types of cancer, including breast cancer [26], and expression is frequently lost in other human epithelial carcinomas [27]. Moreover, other 14-3-3ϵ, ζ, γ, β, θ isoforms have also been identified in cancer [28]. After DNA-damage, 14-3-3σ down-regulated cells fail to maintain a G2/M arrest and undergo a mitotic catastrophe, which results in apoptosis [29]. The reduction of 14-3-3ζ expression induced the G2 arrest, which leads to mitotic catastrophe and increase radio sensitivity [30]. To verify the expressions of identified proteins, we performed an immuno-blotting analysis on 14-3-3σ, 14-3-3ϵ and 14-3-3δ, which have been suggested to be involved in various cancers. Quantification of the protein bands determined that the expression of 14-3-3σ, 14-3-3ϵ and 14-3-3δ were decreased in the vitamin C-treated group compared to the vehicle-treated control group (Figure 4). These data indicated that vitamin C decreased the expression of 14-3-3 isoforms (14-3-3σ, 14-3-3ϵ and 14-3-3δ) in AGS cells. These data suggest that down-regulation of 14-3-3 could be a useful information in therapeutic targets of human gastric cancers.

### Vitamin C down-regulated the cytoskeleton and associated proteins, tropomyosin alpha-3 chain and tropomyosin alpha-4 chain proteins

Tropomyosins are actin-binding proteins that can integrate cell mechanics and signaling essential for cellular migration and invasion. Proteomic studies showed that the expression of tropomyosin changes, which suggest an important role for tropomyosin in maintaining cell shape [31]. In addition, tropomyosins increase filament stiffness, stabilize actin filaments by protecting them against the severing action of gelsolin and cofilin [32]. In our 2 D gel system, the spots corresponding to tropomyosin 3 and tropomyosin 4 showed a decreased expression in the response to vitamin C (Figure 3). These decreased changes in the tropomyosins expression might be coincidental with morphologic changes and migratory characteristics of AGS cells in response to vitamin C.

### Vitamin C up-regulated the antioxidant proteins, peroxiredoxin-4 and thioredoxin domain-containing protein 5

Generally, antioxidant proteins play a pivotal role in the antioxidant defense system and protect the cells from oxidative stress. There are six peroxiredoxins found in mammalians, and peroxiredoxin 4 (PRDX4) is localized in the endoplasmic reticulum (ER) [33]. In the present study, antioxidant and detoxification proteins, like peroxiredoxin-4 and thioredoxin domain-containing protein 5, were over expressed in vitamin C treated AGS cells (Figure 3). Since, vitamin C is an excellent antioxidant that increased antioxidant protein expressions in AGS cells and protects from oxidative stress. To our knowledge there has not been a reported study regarding peroxiredoxin-4 protein expression in human gastric cancer adenocarcinoma AGS cells response to vitamin C. Therefore, a detailed study is necessary regarding the effects of vitamin C on peroxiredoxin-4 protein expressions and the role of peroxiredoxin-4 protein in tumorigenesis of gastric cancer.

### Vitamin C altered the proteins involved in protein metabolism

**Figure 5:**
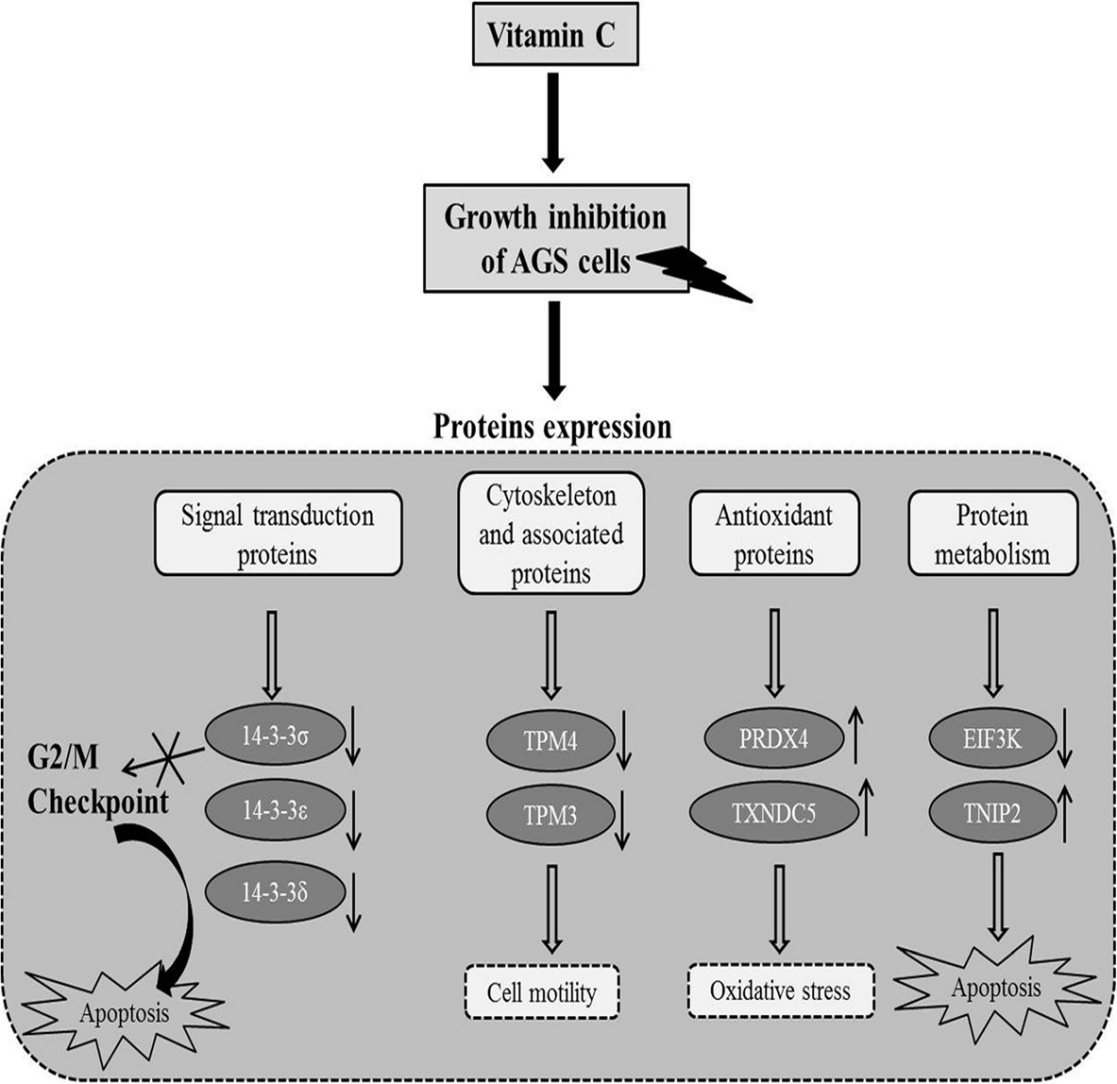
**Flow diagram depicts the modulation of proteins expression due to inhibitory effect of vitamin C in AGS cells.** Vitamin C down-regulated the 14-3-3 isoforms, cytoskeleton and associated proteins (TPM4 and TPM3), and up-regulated antioxidant proteins (PRDX4 and TXNDC5). The down-regulation of 14-3-3 proteins failed to maintain G2/M checkpoint which results in apoptosis. (↓ indicates down-regulation, ↑ indicates up-regulation).

Also, we found that EIF3K was down-regulated in vitamin C-treated AGS cells. A previous study has been reported that the down-regulation of eIF3k attenuating apoptosis in simple epithelial cells [34]. Tumor Necrosis Factor (TNF) Alpha-Induced Protein 3 (also known as A20-binding inhibitor of NF-kappa-B activation 2) or TNFAIP3 is a novel tumor suppressor protein and a key player in the negative feedback regulation of NF-kB signaling in response to multiple stimuli. TNFAIP3 also regulates TNF-induced apoptosis [35]. Moreover, TNFAIP3 induces cell growth arrest and apoptosis, accompanied by down-regulation of nuclear factor-kappa B (NF-kB) activation [36,37]. Presently, TNFAIP3 was up-regulated in vitamin C treated AGS cells. Figure 5 represents the overview of the growth inhibition effect of vitamin C on AGS cells and protein expression patterns. These proteomic results reveal that vitamin C inhibited cell growth, and apoptosis related proteins were involved in promoting and regulating cell death in AGS cells.

## Conclusions

In summary, vitamin C showed strong inhibitory effect on AGS cell growth at pharmacological concentrations (300 μg/mL), and 20 differentially expressed proteins were identified in AGS cells after exposure to vitamin C by using 2-DE and MADLI-TOF analysis. In particular, proteins involved in signal transduction 14-3-3σ, 14-3-3ϵ and 14-3-3δ, and cytoskeletal proteins tropomyosin alpha-3 chain and tropomyosin alpha-4 chain were down-regulated; Peroxiredoxin-4 was up-regulated in vitamin C-treated AGS cells compared with the control. Further, the expressions of 14-3-3 isoforms (14-3-3σ, 14-3-3ϵ and 14-3-3δ) were verified with a Western blot analysis. The findings of this study suggest that vitamin C could inhibit AGS cell growth, alter the apoptosis related proteins, and might be helpful to understand the molecular mechanism of vitamin C ’s anti-tumor effect in AGS cells.

## Competing interests

We declare that there are no conflicts of interests.

## Authors’ contributions

Conceived and designed the experiments: AN and GS-K; Performed the experiments: AN and HS-P; Analyzed the data: AN, HS-P and KI-P; Statistical analysis: JA-K, GE-H, SR-K and JZ; Wrote the manuscript: AN; Drafting and critical review of manuscript for important intellectual content: EH-K, WS-L and CK-W. All authors read and approved the final manuscript.
